# Gluten-Free Diet in Prisons in Poland: Nutrient Contents and Implementation of Dietary Reference Intake Standards

**DOI:** 10.3390/nu12092829

**Published:** 2020-09-16

**Authors:** Aureliusz Kosendiak, Piotr Stanikowski, Dorota Domagała, Waldemar Gustaw

**Affiliations:** 1 Study of Physical Education and Sport, Wroclaw Medical University, 51-601 Wroclaw, Poland; aureliusz.kosendiak@umed.wroc.pl; 2 Department of Plant Food Technology and Gastronomy, Faculty of Food Science and Biotechnology, University of Life Sciences in Lublin, 20-704 Lublin, Poland; waldemar.gustaw@up.lublin.pl; 3 Department of Applied Mathematics and Computer Science, Faculty of Production Engineering, University of Life Sciences in Lublin, 20-612 Lublin, Poland; dorota.domagala@up.lublin.pl

**Keywords:** gluten-free diet, celiac disease, dietary reference intake, prison diets

## Abstract

The gluten-free diet (GFD) requires special attention from nutritionists due to the potential risk of nutrient deficiencies in its users. This risk may be greater when this type of nutrition is implemented in prisons due to the limited possibilities of external control, a low catering budget for meals, and insufficiently defined recommendations regulating nutrition for prisoners. The aim of the present study was to assess the nutritional value of GFD and regular diet meals served in some Polish prisons and to compare the values to the dietary reference intake (DRI) standards. Using a specialized computer program, 7-day menus of both types of diet provided in 10 prisons were analyzed. The percentage coverage of the DRI was calculated based on the recommendations of the Polish National Food and Nutrition Institute. GFD was characterized by lower average contents of energy and 11 out of 14 essential nutrients, i.e., protein, carbohydrates, dietary fiber, starch, ash, sodium, calcium, iron, zinc, folate, and vitamin B_12_. The average content of phosphorus, niacin, and riboflavin in the gluten-free diet was higher than that in the regular diet. It was shown that the meals in GFD and the regular diet did not provide the recommended amounts of calcium (38 and 44% DRI, respectively), vitamin D (29 and 30% DRI), vitamin C (86 and 76% DRI), and folate (51 and 56% DRI). In turn, the supply of sodium, phosphorus, copper, and vitamins A and B_6_ substantially exceeded the recommended levels. The results indicate a need for greater quality control of GFD meals served in catering facilities. It is also necessary to develop legal provisions that will regulate more specifically the nutrition for prisoners in terms of an adequate supply of minerals and vitamins.

## 1. Introduction

Gluten is a general term given to the following fractions of protein: gliadins, glutenins, hordein, and secalin. These protein fractions are found in four grains, i.e., wheat, rye, barley, and triticale. Oats are inherently gluten-free but may be contaminated with wheat during growing or processing [1]. The ingestion of gluten can trigger an array of conditions; they are designated by a broader term “gluten-related disorders”. They are divided into disorders with autoimmune pathogenesis, including celiac disease (CD), disorders characterized by allergic mechanisms, which include wheat allergy, and the controversial non-celiac gluten sensitivity, whose causes are neither autoimmune nor allergic in nature [2].

CD is a common chronic immune-mediated small bowel enteropathy resulting from gluten exposure in genetically susceptible individuals [3]. It is generally acknowledged that about 1% of the general population have CD [4]. In the US population, a higher proportion of persons living at latitudes of 35° North or greater have CD or avoid gluten than persons living south of this latitude, independent of the race or ethnicity, socioeconomic status, or body mass index [5]. Unfortunately, there are no data about the prevalence rate of CD and other gluten-related disorders in the health statistics reports on prisoners [6,7,8].

Currently, the only effective treatment available for CD is a strict life-long gluten-free diet(GFD), since it leads to resolution of intestinal and extraintestinal symptoms, negativity of autoantibodies, and regrowth of intestinal villi. In addition, the diet exerts a partial protective effect on several complications. However, these crucial advantages are accompanied by some disadvantages, including a negative impact on the quality of life, psychological problems, fear of involuntary/inadvertent gluten contamination, increased cardiovascular risk, and frequent severe constipation [9]. Gluten-free food products are substantially more expensive than regular equivalents. Replacement of commonly consumed cereal staple foods in GFDs with gluten-free equivalents may be associated with an increased supply of fat, saturated fatty acids, salt, and sugar [10]. GFD may lead to possible nutrient deficiencies of fiber [11,12], folate [11,12,13,14], vitamin D [11,14,15], calcium [11,12,14,16], magnesium [11,12,16], iron [12,14], zinc [16], selenium [16], and iodine [14]. To increase the supply of nutrients, it is recommended to include legume and pseudo-cereal products (especially amaranth, quinoa, and soybeans) in GFD. They are a better source of fat, fiber, high-quality protein, and minerals than the frequently served corn and rice [17].

The aim of the present study was to assess the nutritional value of gluten-free and regular diet meals served in some Polish prisons and to compare the values to the dietary reference intake standards. 

## 2. Materials and Methods

### 2.1. General Information

The study was approved by the Director-General of the Prison Service in Poland on 3 December 2018. Next, a request for access to the menus was sent in an electronic or paper form to all the institutions. In total, 88 prisons responded to the request. Ten independent prisons, all serving gluten-free diet, were selected for the investigations, i.e., detention centers in Gdańsk, Poznań, Suwałki, and Wrocław and prisons in Dębica, Grądy Woniecko, Iława, Nysa, Strzelce Opolskie (No. 2), and Włodawa. Most of the institutions were male prisons, whereas three facilities were female and male prisons. All prisons were designed for adults. 

### 2.2. Analysis of Energy and Nutrient Content

The analysis involved 7-day GFD menus (from different seasons) and 7-day regular diet menus (from different seasons) provided by each prison in 2018. The regular diet was served to all healthy adult prisoners, while the GFD was prescribed by medical staff [18]. In the study, 140 all-day menus consisting of breakfast, lunch, and supper were analyzed. A typical GFD breakfast usually consisted of puffed rice cakes, margarine, sandwich meats, jam, and an apple/vegetable. Various types of soup and a dish composed of meat, potatoes/white rice, and side salad were served for lunch. Supper mostly included white rice/puffed rice cakes, margarine, and sandwich meats/cottage cheese. With each meal, prisoners made tea themselves. The calculations did not include food that prisoners were able to buy at least three times a month in the prison canteen or food parcels that prisoners received once a month from their relatives [19].

The quantitative analysis was carried out with the use of specialized software DietetykPro (DietetykPro, Wrocław, Poland), which mainly incorporates Polish Food Composition databases developed at the National Food and Nutrition Institute in Warsaw [20] and the database of the United States Department of Agriculture [21]. All food products specified in the menus and inventory reports were analyzed. The inventory reports included names of the food products and their quantity in kilograms/liters used in the kitchen to prepare all meals. Ready meals included in the software database were not taken into account in the analysis. The study involved assessment of 31 parameters of daily food rations: energy value, total protein, total fat, total carbohydrate, dietary fiber, sucrose, starch, cholesterol, fatty acids (saturated, monounsaturated, polyunsaturated), ash, minerals (sodium, potassium, calcium, phosphorus, magnesium, iron, zinc, copper), and vitamins A (as retinol activity equivalents), retinol, B_1_, B_2_, niacin, B_6_, B_12_, C, D, E, and folate (as dietary folate equivalents). The results took into account averaged technological losses caused by heat treatment: folate and vitamin C, 50%; vitamin B_1_, 30%; vitamin B_6_, 25%; vitamins A, E, retinol, and niacin, 20%; and other parameters, 10%. Next, the percentage of the dietary reference intake (DRI) was calculated based on nutrition standards for the Polish population [22]. Since approximately 57% of male prisoners in Poland in 2019 were in the age range of 31–50 [8], the calculations were based on recommendations for this group. For calculation of energy requirement, the physical activity level (PAL) of 1.4 was adopted.

Next, the DietetykPro and Microsoft Excel software was used to analyze the consumption of food groups. The classification of the food groups and subgroups was based on the Polish Food Composition databases [20]. Certain modifications in the classification have been introduced for better presentation of the differences between the analyzed diets. In the “cereal products” group, a sub-group “puffed rice cakes” has been added. The “vegetables and vegetable products” group has been supplemented with a “starchy roots” sub-group, and the “legumes” sub-group has been removed and analyzed as a separate “legumes” group.

### 2.3. Statistical Analysis

The statistical analysis was carried out using the Microsoft Excel 2020 and Statistica 13.1 program (StatSoft, Cracow, Poland). Welch’s test was used to check whether the type of diet had an effect on the average content of the analyzed nutrients, energy, and daily consumption of the food groups. In the next step, 95% confidence intervals for differences between the means of 15 components significantly differing between the gluten-free and regular diets were determined. Similarly, such intervals were calculated for the daily consumption of seven food groups which were differed significantly in both types of diet. 

## 3. Results

### 3.1. Energy and Macronutrients

The daily energy supply met the recommendations of the Polish National Food and Nutrition Institute in the case of GFD, but exceeded the recommended values by 108 kcal in the regular diet (Table 1). The supply of saturated fatty acids (SFA) was 24.5 g in the case of GFD and 26.3 g in the case of the regular diet, which exceeded the recommended values. SFAs covered approx. 9.0% of total energy intake. 

### 3.2. Micronutrients

The average supply of micronutrients in the daily food ration is presented in Table 2. In comparison with the recommendations, excess consumption of three minerals, i.e., sodium, phosphorus, and copper, was recorded in both diets. A particularly high supply was recorded in the case of sodium. The consumption of potassium slightly exceeded the recommended values. The supply of magnesium, iron, and zinc was close to the reference values. The lowest supply of all minerals was recorded for calcium. Its intake in GFD was 378.7 mg, which covered 38% of DRI. The intake of this element in the regular diet was 440.3 mg, which represented 44% of DRI. 

The content of vitamins A and B_6_ in the analyzed menus substantially exceeded the recommended values. In turn, the supply of vitamin D in both diets was very low, i.e., 4.3 and 4.5 µg, respectively. This only covered 29% of the recommended values in GFD and 30% in the regular diet. Both diets were characterized by a low intake of folate, covering approximately half of the DRI value.

### 3.3. Analysis of the Menus by Types of Diet

The gluten-free and regular diets differed statistically significantly in the content of energy and 14 nutrients: protein, carbohydrates, dietary fiber, starch, ash, sodium, calcium, phosphorus, iron, zinc, riboflavin, niacin, folate, and vitamin B_12_. For the differences between the mean levels of the essential ingredients in the regular diet and GFD, 95% confidence intervals were determined (Table 3). GFD was characterized by lower average contents of energy and 11 of the 14 essential nutrients, i.e., protein, carbohydrates, dietary fiber, starch, ash, sodium, calcium, iron, zinc, folate, and vitamin B_12_. The average content of phosphorus, niacin, and riboflavin in GFD was higher than in the regular diet. 

### 3.4. Analysis of the Food Group Consumption

In both types of diet, no consumption of products from the groups and subgroups “frozen fruits”, “fruit, dried”, “nuts”, “seeds”, and “beverages” was recorded (Table 4). Products from the subgroup “mushrooms” were served in only three prisons. In GFD, no products from the subgroups “pasta” (including gluten-free pasta), “breads and rolls” (including gluten-free breads and rolls) were served, and “legumes” were noted in only one object. The regular diet was characterized by no consumption of products from the subgroup “puffed rice cakes”.

The regular diet and GFD differed significantly in terms of the consumption of products from the following food groups and subgroups: “cereal products”, “groats”, “vegetable products”, “fish, fish products, and seafood”, “fruits and fruit products”, “fruit, raw”, and “other products” (Table 5). In GFD, the consumption of products from the groups “groats”, “fruits and fruit products”, and “fruit, raw” was significantly higher than in the regular diet and significantly lower in the case of the other groups (“cereal products”, “vegetable products”, “fish, fish products and seafood”, and “other products”) than in the regular diet (Table 5).

## 4. Discussion

The investigations conducted by our team revealed inadequate quality of meals served in the Polish prisons. Compared to the regular diet, GFD was characterized by a significantly lower average level of energy and 11 nutrients: protein, carbohydrates, dietary fiber, starch, ash, sodium, calcium, iron, zinc, folate, and vitamin B_12_. The mean content of phosphorus, niacin, and riboflavin was higher in GFD than in the regular diet.

The implementation of GFD involves exclusion of many food products. Wheat or mixed bread was found to be the basic food served for breakfast and supper in almost all diets available in the Polish prisons. It was mainly replaced with puffed rice cakes in the GFD meals. Therefore, the cost of GFD breakfast and supper was high, since puffed rice cakes were up to 10 times more expensive than bread, as shown by the inventory reports. Hence, prison meal planners tended to limit the amount of these products served for breakfast and supper even twice in comparison with the ration of bread served in these meals in the other diets. The difference in the consumption of cereal products between the regular diet and GFD, i.e., 243.66 g, probably had an impact on the supply of energy, protein, and carbohydrates, which was significantly higher in the regular diet. As suggested by Soto et al. [23], the difference in the energy value between GFD and regular diet meals may also be associated with the exclusion of breaded fried foods. Bread, rolls, and bread products contribute substantially to the supply of many nutrients. In the average Polish diet, these products provide 48.6% of manganese, 36.3% of carbohydrates, 35.4% of fiber, 24.9% of iron, 22.1% of copper, 21.1% of zinc, 21% of magnesium, and 20.7% of folate [24]. Therefore, the exclusion of bread from GFD may have resulted in the considerably lower consumption of such nutrients as fiber, iron, zinc, and folate, in comparison with the regular diet.

The average energy value was 2405.5 kcal/day in the GFD menus and 2708.0 kcal/day in the regular diet. The energy value in the latter diet was higher than the nutritional standards recommended for the Polish population (2100–2600 kcal) [22]. As specified by the regulations on nutrition for prisoners in Poland, meals should provide at least 2600 kcal [18]. In our opinion, these recommendations require personalization, which is supported by the varied physical activity [25] and excessive body weight in Polish prisoners [26].

The analysis of the menus did not show any disturbing observations regarding the supply of macronutrients in the diet. Only the consumption of SFAs exceeded the recommended values in both analyzed diets. However, no significantly higher consumption of this ingredient was detected in GFD, which is usually observed in patients with celiac disease [27]. This may be related to the higher SFA content in GF products than in their counterparts [28]. The lower supply of fiber in GFD compared to the regular diet indicated by the present results is in agreement with values reported by other authors [16,28,29]. The consumption of dietary fiber in GFD covered DRI, although it was significantly lower than in the regular diet. This may be related to the significantly higher consumption of groats, fruit, and fruit products in this diet compared to GFD, which we showed in the analysis of the consumption of the food groups. It is recommended that GFD meals should be enriched with fiber and minerals through consumption of legumes and pseudocereals [17]. The analysis conducted in the study showed very low consumption of legumes, which in the case of GFD were included in the menu in only one prison, whereas no pseudocereals were served. The main ingredient of lunch was white rice, while brown rice, which provides more fiber and many other health-beneficial food components [30], was not served at all. The analyzed GFD diets did not include oat, which is nutritious and a good source of fiber and can be safely consumed by patients with CD [31]. However, according to the recommendations of the Polish Association of People with Celiac Disease and the Gluten-Free Diet, oats and oat products in Poland are highly contaminated with gluten and therefore should not be used in the GFD [32].

To our knowledge, the large difference in the supply of sodium between the two analyzed diets was associated with the fact that the meal planners followed invalid provisions regulating the issue of nutrition in prisons in Poland [33]. These regulations recommended that the daily salt intake in therapeutic diets should be limited to 6 g per day. The analysis of the menus and inventory reports demonstrated that the regular diet and GFD were planned to contain 3 to 11 g and 0 to 3 g of table salt per day, respectively. We reported high levels of salt additions to prison meals in our previous investigations as well [34]. Besides the lower addition of salt to the dishes, the sodium content in GFD may also have been influenced by the exclusion of bread from the diet. Bread is a source of 17.5% of sodium in the average diet of the Polish population [24] and 23% of sodium in the diet of Polish hypertension patients [35]. As far as other minerals are concerned, a significantly low calcium intake was noted in both diets. The low calcium supply is reflected in the level of consumption of the respective food groups. The average consumption of milk and dairy products, which are the most important source of this mineral, was only 61.96 g in GFD and 56.14 g in the regular diet. An adequate supply of this mineral is particularly important in CD patients, due to the malabsorption of the nutrient, which may lead to development of bone diseases [36]. Individuals with undiagnosed and untreated CD are at the highest risk of malabsorption [37]. GFD was characterized by a significantly lower supply of calcium than the regular diet. There are inconsistent data showing differences in the supply of this component between GFD and regular diets. The results reported by Dall’Asta et al. [27] confirm our observations, whereas Wild et al. [16] suggest that patients with GFD may consume higher amounts of calcium than those with a non-GFD diet. There are also considerable differences in the supply of this nutrient to CD patients depending on their sex and age [38]. Due to the high supply of phosphorus, the menus analyzed in the present study had a very unfavorable Ca:P ratio, i.e., 0.26:1 in GFD and 0.32:1 in the regular diet. The correct ratio of these two minerals is 1.5:1 [39]. 

The supply of vitamins in both diets was especially high in the case of vitamins A and B_6_. The consumption of vitamins C, vitamin D, and folate did not meet the recommended intake. An inadequate supply of these nutrients was also observed in other studies on the nutrition of Polish prisoners [34,40,41]. The high supply of vitamin A in the analyzed menus was probably related to the frequent use of margarine (30–90 g per day), which is obligatorily fortified with this vitamin in Poland. The large standard deviations in the case of the vitamin A supply were probably associated with the presence of fried pork liver in many menus for the convicts. The supply of vitamin B_6_ in both diets covered over 200% of DRI. It is generally lower in the Polish population, as 16% of males and 36% of females do not consume or supplement its recommended amounts [42]. Vitamin D, the consumption of which in the daily food ration did not cover even half of the recommended amount, is especially important for celiac disease patients. It plays a key role in the regulation of immune response and may have an impact on CD [43]. As shown by research conducted in the USA, only 31% of prisoners have normal levels of this component in blood [44]. The cutaneous synthesis of this vitamin in prisoners may be lower than in the general population due to the limited time spent outdoors. However, deficiencies of this component have even been detected in 90% of individuals imprisoned in places with high sun exposure [45]. In our opinion, the low supply of vitamin C was probably caused by the limitation of the assortment of vegetables to a few cheapest ones, which are not a good source of this vitamin (beetroot, carrots, onions, and potatoes). Similarly, the range of fruit served in the diets sporadically was limited to apples. The infrequent presence of vegetables in the diets probably contributed to the low supply of folate. A significantly lower supply of this nutrient was noted in GFD, which may have been associated with the exclusion of many cereal products from this diet.

Analyses of the present results should take into account that the quality of GFD meals served in prisons may be influenced by the low catering budget allowance. This was evidenced by the results of our previous investigations of the nutrition for Polish prisoners, which demonstrated that the limited financial means resulted in a large reduction in the range of fruit and vegetables served in prison meals [34]. The mean all-day purchase-only cost of prison-provided meals and beverages in the GFD was 6.65 ± 0.61 PLN (1.49 ± 0.14 EUR). In Polish supermarkets, this is a purchase price of only 250 g of gluten-free bread or 300 g of gluten-free pasta. Gluten-free (GF) products are more expensive than their standard equivalents [46]. As shown by some data, they may be on average 159% more expensive than regular products in the UK [10] and from 22 to even 334% more expensive in Greece [47]. Probably because of these costs, the menus in all prisons did not include certified GF products; instead, the meals were based on naturally GF products. Both groups of GF products, however, may contain various levels of gluten. Verma et al. [48] showed that its allowable value (20 ppm) was exceeded in 9% of GF products available in stores in Italy.

GFD requires special attention from nutritionists and kitchen staff not only in terms of the nutritional value of the meals served. The difficulties in providing this type of diet in food service establishments are associated with an appropriate supply of GF products, storage of these products, production processes, tools, and processing methods [49]. It has been shown that contamination of kitchen utensils or food-contact surfaces with gluten in school kitchens is promoted by the use of non-protease detergents, lack of rinsing with water immediately before use, storage in open containers, and washing in dishwashers (compared to manual washing) [50]. Therefore, there is a need to investigate GFD served in prisons, with focus on the aspect of proper meal processing in this diet.

### Limitations

It should be emphasized that the present results are based solely on analysis of the menus, but they do not include food that prisoners can buy and receive from families. Another limitation in the results is the lack of knowledge of whether all prisoners consume the same portions of meals. This is related to the complicated prison hierarchy and confinement in cells.

## 5. Conclusions

The results of our research indicate that GFD meals in Polish prisons provide significantly lower amounts of many micronutrients than regular diet meals. This is caused by the exclusion of gluten-containing food from this diet, which is an important source of, e.g., fiber, iron, zinc, and folates. Both analyzed types of diets exhibited excess levels of SFA, sodium, calcium, phosphorus, copper, and vitamins (A, B_6_, C, D and folates). The results regarding the supply of nutrients necessitate action from the Central Board of the Prison Service aimed at introduction of external controls and improvement of the quality of meals. There is also a need for a comprehensive discussion on the possibility of supplementation of prisoners, especially with vitamin D. Undoubtedly, the quality of meals is related to the limited financial resources in Polish prisons. This makes it difficult to plan balanced meals by prison nutritionists, especially in the case of GFD meals, which are more expensive.

## Figures and Tables

**Table 1:** Energy and macronutrients provided in prisons (*n* = 10) menus per person per day and the age-specific dietary reference intake (DRI).

Observed Component	Recommended	Gluten-Free Diet	Regular Diet
		Mean ± SD	Mean ± SD
Energy (kcal)	2100–2600 (EER)	2405.5 ± 355.6	2708.0 ± 258.6
Protein (g)	50–77 (RDA)	82.3 ± 10.5	90.6 ± 15.6
Fat (g)	70–87 ^1^	72.4 ± 21.2	79.0 ± 18.2
SFA (g)	max. 17.6–21.1	24.5 ± 11.9	26.3 ± 8.9
MUFA (g)	N.A.	28.6 ± 9.1	31.0 ± 7.3
PUFA (g)	N.A.	15.4 ± 6.5	16.2 ± 5.3
Cholesterol (mg)	N.A.	244.0 ± 128.6	243.7 ± 106.4
Carbohydrates (g)	130 (RDA)	370.7 ± 57.6	429.5 ± 40.9
Starch (g)	N.A.	149.4 ± 48.0	293.4 ± 37.4
Sucrose (g)	N.A.	53.7 ± 18.1	52.5 ± 18.6
Fiber (g)	25 (AI)	30.0 ± 6.4	37.3 ± 6.4

^1^ 30% of energy from fats; EER estimated energy requirement; RDA recommended dietary allowance; SFA saturated fatty acids; MUFA monounsaturated fatty acids; PUFA polyunsaturated fatty acids; AI adequate intake; N.A. not available.

**Table 2:** Micronutrients provided in prisons (*n* = 10) menus per person per day and the age-specific dietary reference intake (DRI).

Observed Component	Recommended	Gluten-Free Diet		Regular Diet	
		Mean ± SD	% of DRI	Mean ± SD	% of DRI
Ash (g)	N.A.	18.2 ± 3.6	N.A.	29.7 ± 4.3	N.A.
Sodium (mg)	1500 (AI)	3073.9 ± 910.8	205	7727.0 ± 1569.7	515
Potassium (mg)	3500 (AI)	4892.6 ± 1143.5	140	4628.8 ± 1049.5	132
Calcium (mg)	1000 (RDA)	378.7 ± 163.2	38	440.3 ± 128.5	44
Phosphorus (mg)	700 (RDA)	1452.0 ± 172.8	207	1377.6 ± 218.3	197
Magnesium (mg)	420 (RDA)	410.3 ± 76.2	98	397.4 ± 76.4	95
Iron (mg)	10 (RDA)	11.6 ± 2.7	116	16.1 ± 4.4	161
Zinc (mg)	11 (RDA)	12.2 ± 2.4	111	13.4 ± 2.3	122
Copper (mg)	0.9 (RDA)	1.7 ± 0.3	189	1.8 ± 0.4	200
Vitamin A (µg)	900 (RDA)	2776.3 ± 1319.0	308	2339.3 ± 2437.9	260
Retinol (µg)	N.A.	279.7 ± 211.2	N.A.	681.0 ± 2161.3	N.A.
Vitamin D (µg)	15 (AI)	4.3 ± 3.3	29	4.5 ± 4.3	30
Vitamin E (mg)	10 (AI)	12.0 ± 3.9	120	11.3 ± 3.5	113
Vitamin B_1_ (mg)	1.3 (RDA)	1.4 ± 0.4	108	1.5 ± 0.4	115
Vitamin B_2_ (mg)	1.3 (RDA)	1.3 ± 0.4	100	1.1 ± 0.7	85
Niacin (mg)	16 (RDA)	22.3 ± 4.9	139	19.7 ± 5.0	123
Vitamin B_6_ (mg)	1.3 (RDA)	2.9 ± 0.5	223	3.0 ± 1.8	231
Vitamin B_12_ (µg)	2.4 (RDA)	2.1 ± 1.1	88	4.3 ± 5.5	179
Vitamin C (mg)	90 (RDA)	77.4 ± 33.9	86	68.1 ± 26.9	76
Folate (mg)	400 (RDA)	204.2 ± 52.2	51	222.2 ± 47.5	56

AI adequate intake; RDA recommended dietary allowance; N.A. not available.

**Table 3:** Lower and upper endpoints of a 95% confidence interval for the difference between mean regular and gluten-free diet.

Observed Component	${\bar{x}}_{r}-{\bar{x}}_{gf}$	Lower Endpoint	Upper Endpoint
Energy (kcal)	302.49	198.58	406.40
Protein (g)	8.29	3.84	12.74
Carbohydrates (g)	58.78	42.09	75.47
Fiber (g)	7.34	5.21	9.47
Starch (g)	143.95	129.56	158.34
Ash (g)	11.48	10.15	12.82
Sodium (mg)	4653.14	4224.23	5082.04
Calcium (mg)	61.66	12.57	110.75
Phosphorus (mg)	−74.40	−140.21	−8.59
Iron (mg)	4.48	3.26	5.71
Zinc (mg)	1.28	0.48	2.08
Riboflavin (mg)	−0.23	−0.41	−0.05
Niacin (mg)	−2.59	−4.24	−0.95
Folate (mg)	18.06	1.39	34.73
Vitamin B_12_ (µg)	2.21	0.89	3.52

${\bar{x}}_{r}$—mean value for regular diet, ${\bar{x}}_{gf}$—mean value for gluten-free diet.

**Table 4:** Distribution of food group and sub-group consumption (g/day).

Food Groups and Sub-Groups	Gluten-Free Diet		Regular Diet	
	Mean	SD	Mean	SD
**Cereal products**	221.34	58.40	465.00	34.42
Grains, flours and starches	14.30	23.06	13.30	3.80
Groats	100.07	73.40	37.02	9.88
Pasta	0.00	0.00	22.89	9.45
Breads and rolls	0.00	0.00	391.17	30.66
Breakfast cereals	1.52	2.87	0.98	1.91
Puffed rice cakes	105.45	16.96	0.00	0.00
**Vegetables and vegetable products**	919.39	153.68	850.51	106.96
Vegetables, raw and boiled	402.27	91.11	326.52	68.22
Frozen vegetables	27.64	28.62	10.62	12.24
Vegetable products	3.75	4.56	77.70	44.63
Mushrooms	1.18	3.33	3.23	6.27
Starchy roots	484.55	104.96	432.45	77.71
**Legumes**	1.79	5.05	19.72	6.86
**Fruits and fruit products**	346.72	285.06	88.79	66.81
Fruit, raw	344.77	251.32	62.70	62.09
Frozen fruits	0.00	0.00	0.00	0.00
Fruit, dried	0.00	0.00	0.00	0.00
Fruit products	54.64	40.09	26.09	14.10
**Nuts**	0.00	0.00	0.00	0.00
**Seeds**	0.00	0.00	0.00	0.00
**Milk and milk products**	61.96	39.05	56.14	27.77
**Meat and meat products**	241.52	49.64	222.92	40.74
**Fish, fish products and seafood**	10.71	12.27	38.32	15.69
**Eggs**	15.73	22.94	10.86	7.27
**Fats and oils**	64.25	26.26	46.31	14.31
**Sugar and confectionery**	25.43	18.49	32.18	10.65
**Beverages**	0.00	0.00	0.00	0.00
**Other products**	14.52	9.10	38.12	9.86

Food groups are bolded in the table.

**Table 5:** Lower and upper endpoints of a 95% confidence interval for the difference between mean regular and gluten-free diet.

Food Groups and Sub-Groups	${\bar{x}}_{R}-{\bar{x}}_{GF}$	Lower Endpoint	Upper Endpoint
Cereal products	243.66	191.10	296.21
Groats	−63.05	−124.53	−1.57
Vegetable products	73.95	36.59	111.30
Fish, fish products and seafood	27.61	12.42	42.79
Fruits and fruit products	−257.93	−497.89	−17.96
Fruit, raw	−282.07	−493.83	−70.31
Other products	23.60	13.42	33.78

${\bar{x}}_{R}$—mean value for regular diet, ${\bar{x}}_{GF}$—mean value for gluten-free diet.
